# Global signal topography of the depressive syndrome in bipolar disorder

**DOI:** 10.1192/j.eurpsy.2023.1159

**Published:** 2023-07-19

**Authors:** G. Gulino, A. Scalabrini, M. Paolini, M. Palladini, F. Benedetti

**Affiliations:** 1 Università Vita-Salute San Raffaele; 2 Università di Bergamo; 3 IRCCS San Raffaele Scientific Institute, Milano, Italy

## Abstract

**Introduction:**

Previous findings show that the depressive state is characterized by a peculiar suppression of the resting state functional connectivity (rsFC) anti-correlation between resting-state networks (e.g., Default Mode Network) and task-positive networks (e.g., Sensory-Motor Network) in favor of an abnormal positive rsFC pattern. This suggests a large-scale functional disbalance in adaptively switching the attentional focus from an internal-oriented cognitive modality to an external-oriented processing modality. Yet, according to further evidence, such a functional inversion is primarily driven by the global signal (GS) (i.e., by an abnormal large-scale topographical reconfiguration) in major depressive disorder (MDD). However, it is not clear if similar alterations may affect bipolar disorder (BD) in depressive phase.

**Objectives:**

Investigation of the global topography of the depressive syndrome as a potential transnosographic endophenotype and evaluation of the GS on generating differences between groups.

**Methods:**

We compared large-scale rsFC patterns in a group of healthy controls (HC) (n=70) and a group of patients with BD (n=70) during a depressive episode. In order to investigate the impact of the GS, we further performed all analyses both with and without GS regression (GSR).

**Results:**

Compared to HC, patients with an ongoing major depressive episode exhibit specific resting-state changes that are only observed when analysis is performed without regressing GS. Patients were found to exhibit an (i) abnormally strong GS contribution within an extended cluster comprising regions known to be part of highly interconnected hubs (i.e., *transmodal networks*) and showing functional relations’ core along the cortical midline and a (ii) diminished influence of the GS in correspondence of frontoparietal and occipitotemporal regions. Notably, no traces of such changes -differentiating the global topography of patients from HC- held when applying GSR.

**Conclusions:**

Our results (i) suggest that rsFC alterations detected stem from a global rather than a local source and (ii) corroborate the impact GS can exert on generating within and between-networks differences. Hence, we underline the necessity that future investigations on groups with expected altered topographical distribution include GS within data-analysis and a proper evaluation of its involvement. Nonetheless, our results are in line with previous evidence of altered global topography in MDD. Hence, we interpreted this finding as a benchmark of a whole-brain functional disbalance toward self-oriented cognition characterizing the transnosographic depressive syndrome.

**Disclosure of Interest:**

None Declared

